# Incidence of Subsequent Injuries Associated with a New Diagnosis of Benign Paroxysmal Positional Vertigo and Effects of Treatment: A Nationwide Cohort Study

**DOI:** 10.3390/jcm13154561

**Published:** 2024-08-05

**Authors:** Jhen-Jie Mao, Hung-Che Lin, Shih-Tsang Lin, Po-Cheng Lin, Ching-Hsiang Chang, Wu-Chien Chien, Chi-Hsiang Chung, Ying-Jiin Chen, Jeng-Wen Chen

**Affiliations:** 1 Department of Medical Education and Research, Cardinal Tien Hospital and Fu Jen Catholic University, New Taipei City 23148, Taiwan; 407510191@m365.fju.edu.tw (J.-J.M.); 407510098@m365.fju.edu.tw (P.-C.L.); 407510268@m365.fju.edu.tw (C.-H.C.); 2 Department of Otolaryngology–Head and Neck Surgery, Tri-Service General Hospital, National Defense Medical Center, Taipei 114202, Taiwan; lhj50702@mail.ndmctsgh.edu.tw; 3 Department of Otolaryngology–Head and Neck Surgery, Cardinal Tien Hospital and Fu Jen Catholic University, New Taipei City 23148, Taiwan; tsang4lin@hotmail.com; 4 Department of Otolaryngology–Head and Neck Surgery, National Taiwan University Hospital, Taipei 100225, Taiwan; 5 Department of Medical Research, Tri-Service General Hospital, National Defense Medical Center, Taipei 114202, Taiwan; chienwu@mail.ndmctsgh.edu.tw (W.-C.C.); g694810042@mail.ndmctsgh.edu.tw (C.-H.C.); 6 School of Public Health, National Defense Medical Center, Taipei 114202, Taiwan; 7 Department of Emergency Medicine, Cardinal Tien Hospital, New Taipei City 23148, Taiwan; 8 Department of Medical Management, Graduate Institute of Business Administration, Fu Jen Catholic University, New Taipei City 24205, Taiwan; 9 Department of Education and Research, Cardinal Tien Junior College of Healthcare and Management, New Taipei City 231038, Taiwan

**Keywords:** benign paroxysmal positional vertigo, injury, canalith repositioning therapy, fall, traffic injury

## Abstract

**Background/Objectives**: Benign paroxysmal positional vertigo (BPPV) is the most common cause of recurrent vertigo and the most common peripheral vestibular disorder. It is characterized by intense vertigo triggered by head and position changes. This study investigates the risk of subsequent injury in BPPV patients and the effects of treatment. **Methods**: A population-based retrospective cohort study was conducted using data from the Longitudinal Health Insurance Database 2005 in Taiwan. Patients with and without BPPV were identified between 2000 and 2017. The study outcomes were diagnoses of all-cause injuries. The Kaplan–Meier method determined the cumulative incidence rates of injury in both cohorts, and a log-rank test analyzed the differences. Cox proportional hazard models calculated each cohort’s 18-year hazard ratios (HRs). **Results**: We enrolled 50,675 patients with newly diagnosed BPPV and 202,700 matched individuals without BPPV. During follow-up, 47,636 patients were diagnosed with injuries (13,215 from the BPPV cohort and 34,421 from the non-BPPV cohort). The adjusted HR for injury in BPPV patients was 2.63 (95% CI, 2.49–2.88). Subgroup analysis showed an increased incidence of unintentional and intentional injuries in BPPV patients (aHR 2.86; 95% CI, 2.70–3.13 and 1.10; 95% CI, 1.04–1.21, respectively). A positive dose–response relationship was observed with increasing BPPV diagnoses. Treatment with canalith repositioning therapy (CRT) or medications reduced the risk of injury slightly but not significantly (aHR, 0.78; 95% CI, 0.37–1.29, 0.88; 95% CI, 0.40–1.40, respectively). **Conclusions**: BPPV is independently associated with an increased risk of injuries. CRT or medications have limited effects on mitigating this risk. Physicians should advise BPPV patients to take precautions to prevent injuries even after treatment.

## 1. Introduction

Benign paroxysmal positional vertigo (BPPV), one of the most common causes of peripheral vertigo [1,2], is characterized by brief episodes of vertigo attack lasting from seconds to minutes that are triggered by head and position changes [1,2,3,4,5]. According to a population-based retrospective cohort study [6], the lifetime prevalence of BPPV is 2.4%, the mean age of onset was 49.4 years, and the ratio of women to men was between 2:1 and 3:1. Notably, the 1-year prevalence of BPPV in the population older than 60 years was almost seven times higher than that in the age group of 18–39 years [6,7].

Many factors are associated with BPPV, including advanced age, being a woman, head trauma, other ear diseases, migraine, diabetes, osteoporosis, and intubation [6]. The mechanism of BPPV is caused by canalolithiasis or cupulolithiasis. The widely accepted theory regarding canalolithiasis is that the presence of otolith debris in the semicircular canal disrupts the endolymph flow, causing vertigo [2,8,9,10,11]. Of the semicircular canals, the posterior semicircular canal is predominantly affected [8]. In cupulolithiasis, otolith debris adheres to the cupula [2]. The canalith repositioning therapy (CRT), a treatment for BPPV, is based on the canalolithiasis theory [2,8].

Most patients with BPPV experience abrupt onset rotational vertigo (86%) events, often lasting less than 1 min. Other symptoms, including oscillopsia (31%), nausea (33%), vomiting (14%), imbalance (49%), awakening due to BPPV (49%), and fear of falling (36%), are also reported [6]. Episodes are triggered by certain head and body movements, such as turning over in bed (85%) and lying down (74%) [6]. Further, during the attacks and between these episodes, patients experience gait disturbance in their daily lives [12,13,14], and these symptoms lead to psychological problems in patients with BPPV [6,15]. Patients with common types of peripheral vestibular diseases (PVD), namely, BPPV, Ménière’s disease, vestibular neuritis, and unspecified peripheral vestibular dizziness, may experience vertigo, nausea, vomiting, and gait instability in daily life [16,17]. Because of the impact of BPPV symptoms on patients’ lives, we speculated that BPPV would increase the risk of subsequent injuries. However, previous studies discussed only one or a few types of injuries associated with PVD (e.g., unintentional injuries). Therefore, this study investigated the risk of all-cause injury after a new diagnosis of BPPV and the effect of BPPV treatment on this risk.

## 2. Materials and Methods

### 2.1. Data Source

We obtained data from the Taiwan Longitudinal Health Insurance Database 2005 (LHID2005), a subset of Taiwan’s National Health Insurance Research Database, containing the information of 2 million randomly selected individuals. This retrospective cohort study was reviewed and approved by the Institutional Review Board of Cardinal Tien Hospital (CTH-110-3-5-027). Written informed consent from participants was waived because data were obtained from a deidentified database. This study adhered to the STROND (the Standards of Reporting of Neurological Disorders) guidelines [18] of research reporting standards.

### 2.2. Study Design and Participants

We included patients with a history of at least 3 diagnoses of BPPV, which was made based on the characteristics of episodic positional vertigo and physical examination such as the Dix–Hallpike maneuver. The diagnoses were made by an otolaryngologist or neurologist in the outpatient or emergency department or upon admission. The patients who received BPPV diagnoses exclusively from non-otolaryngologists or non-neurologists were excluded. The following patients were excluded: (1) those with a history of BPPV before 2000; (2) those with diseases that might cause vertigo or dizziness prior to the index date; (3) those under 18 years of age; (4) those with a diagnosis of injury before the index date; and (5) those with incomplete demographic data. The index date for this cohort was when the patients met the inclusion criteria, which meant the third diagnosis of BPPV if at least 3 times BPPV coding was defined. Subsequently, for each patient in the BPPV cohort, 4 patients without a history of BPPV were selected from the LHID2005 database through propensity score matching based on gender, age, comorbidities, and the year of the index date of the cases. Patients who met the same exclusion criteria as those for patients with BPPV were excluded.

### 2.3. Outcome Measures

Both cohorts were tracked from the index date until the first diagnosis of injury, death, or the end of 2017. Death was defined as the date a patient was removed from the National Health Insurance (NHI) program. Diseases were coded in accordance with the International Classification of Diseases, Ninth Revision, Clinical Modification (ICD-9-CM) and the International Classification of Diseases, Tenth Revision (ICD-10-CM). The codes for the inclusion and exclusion variables are listed in Supplementary Table S1.

We classified injuries as intentional, unintentional, and unknown-intent injuries. Hazard ratios (HRs) for different types of injuries between the BPPV and the non-BPPV cohorts were analyzed. According to previous studies, intentional injuries include violent attacks and self-mutilation or suicide. We want to observe the connection between BPPV and injury. Injury Severity Score (ISS) was used to evaluate the severity of the injury, with an ISS of ≥16 being classified as major trauma.

### 2.4. Potential Confounders

Confounding factors, such as sex, age or age group, location (the patient’s place of residence), urbanization level, insurance premium, season, and level of care (medical center, regional hospital, and combined district hospital and clinic), were adjusted accordingly.

### 2.5. Subgroup Analysis

We performed subgroup analysis to evaluate whether canalith repositioning therapy (CRT, such as Epley maneuver or Barbecue maneuver), medications, and surgeries could affect the risk of injury; medications for BPPV that were considered included antivertigo preparations, antiemetics and antinauseants, other peripheral vasodilators, antimigraine preparations, antipsychotics, anxiolytics, labyrinthine sedatives, antidepressants, and antihistamines for systemic use. Detailed information on the codes for medications and procedures is listed in Supplementary Table S1. Furthermore, we evaluate the injury rate between 1, 2, and 3 visits for BPPV. We assumed that patients diagnosed with BPPV 3 times may be more accurate.

### 2.6. Statistical Analysis

This study used IBM SPSS Statistics, version 22 (Armonk, NY, USA) for all data analysis. We represented continuous data as mean ± standard deviation. The data’s normality was tested using the Kolmogorov–Smirnov test. To compare standard continuous data between those with and without BPPV, we used either the Student’s *t* test or a one-way analysis of variance. The Mann–Whitney *U* test or Kruskal–Wallis test was employed for nonparametric continuous data. We used the chi-square test with Fisher’s exact correction for categorical data. The Kaplan–Meier method was adopted to compute cumulative injury risks, and the log-rank test was employed to assess differences between the BPPV and non-BPPV cohorts. We conducted both univariate and multivariable Cox proportional hazard regression analyses. All of the significant differences between BPPV and non-BPPV groups were incorporated into the regression model to evaluate the adjusted effect of BPPV on injury incidence and identify potential independent predictive variables. The Bonferroni method was used to correct for multiple comparisons in the subgroup analyses. A *p*-value of less than 0.05 was considered statistically significant. The Cox regression analyses provided hazard ratios (HRs) and confidence intervals (CIs) to estimate relative risk.

## 3. Results

### 3.1. Baseline Characteristic

The baseline and demographic patient characteristics of the BPPV and non-BPPV cohorts are shown in Table 1. The average age of the participants was 60.11 ± 15.84 years. After propensity score matching, no significant differences between the BPPV and non-BPPV cohorts in terms of sex, age, or comorbidities were found. However, noticeable differences were evident in location, urbanization level, insurance premium, and degree of treatment. The subsequent multivariable regression analyses adjusted for these potential confounding factors. The average time interval from diagnosis of BPPV to treatment of CRT or medications and from treatment to injury in the BPPV cohort was 1.89 ± 1.66 and 6.29 ± 5.14 years, respectively. For the BPPV and non-BPPV cohorts, the average follow-up period was 14.02 and 14.86 years, respectively (Supplementary Table S2). The characteristics of the BPPV and non-BPPV groups at the end of the follow-up are shown in Supplementary Table S3.

### 3.2. Cumulative Risk of Injury in Two Groups

**Figure 1:**
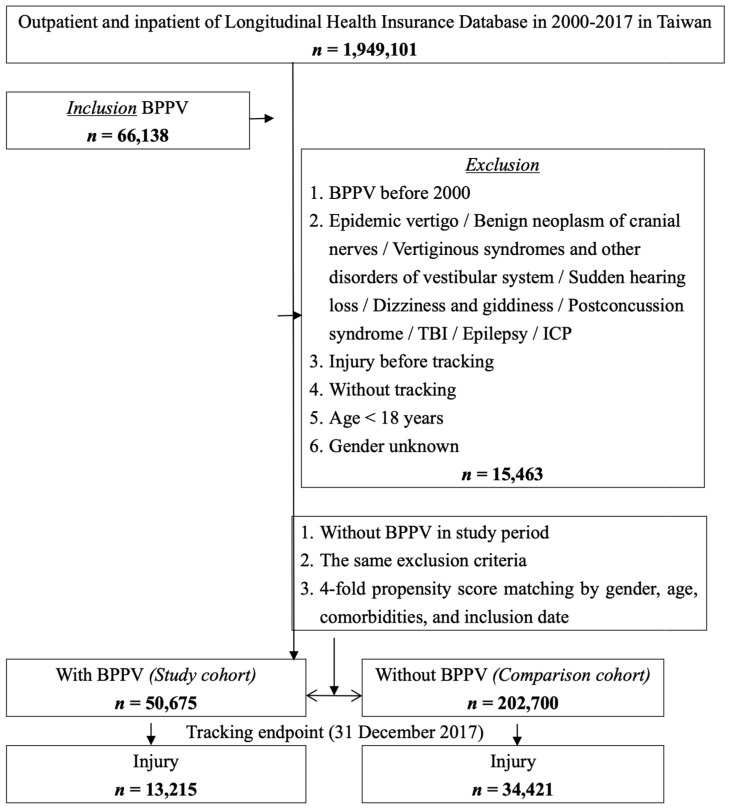
Flowchart of patient selection from the LHID2005. BPPV, benign paroxysmal positional vertigo; TBI, traumatic brain injury; ICP, infantile cerebral palsy.

Of the 1,949,101 patients with outpatient or inpatient records from the LHID2005 claims data from 2000 to 2017 (Figure 1), 50,675 patients with BPPV and 202,700 propensity-score-matched individuals without BPPV were included. At the end of follow-up, 13,215 (26.08%) of the 50,675 patients with BPPV and 34,421 (16.98%) of the 202,700 controls sustained an injury of any type (Table 2, *p* < 0.001). This manifests a higher incidence of subsequent injury in the BPPV group.

**Figure 2:**
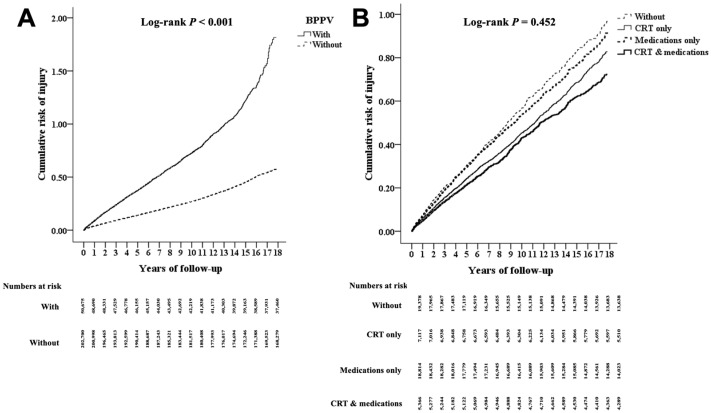
Results of Kaplan–Meier and log-rank test analysis for cumulative risk of all-cause injuries stratified by (**A**) benign paroxysmal positional vertigo, BPPV, and (**B**) treatment with and without canalith repositioning therapy (CRT) or medications. There was a statistically significant difference between the experiment and control groups (significant *p*-value was <0.05).

In Figure 2A, compared with the non-BPPV cohort, the Kaplan–Meier analysis demonstrated a significantly increased risk of injuries in the BPPV cohort over the 18-year follow-up period (log-rank test; *p* < 0.001). Supplementary Table S4 lists the factors associated with injury by the end of follow-up in the Cox regression analysis. In the BPPV cohort, the crude HR of injury was 2.98 (95% CI, 2.56–3.39; *p* < 0.001); after we adjusted for age, gender, comorbidities, insurance premium, geographic location, urbanization level, and level of care, the adjusted HR was 2.63 (95% CI, 2.49–2.88; *p* < 0.001). As shown in Supplementary Table S5, patients with BPPV had a higher risk of injury than the controls in subgroups stratified by gender, age group, insurance premium, comorbidities, urbanization level, geographic location, and level of care. Sensitivity analyses using different time points and definitions of the comorbidities including those in the baseline, at the endpoint and during the study period showed that the adjusted HRs were 2.29 (95% CI: 2.16–2.50, *p* < 0.001), 2.63 (95% CI: 2.49–2.88, *p* < 0.001), and 2.48 (95% CI: 2.34–2.71, *p* < 0.001), respectively (Supplementary Table S6). Subgroup analysis of the incidence of injuries in patients with ≥1, ≥2, and ≥3 times BPPV diagnosis demonstrated a positive dose–response relationship. Those patients with ≥1, ≥2, and ≥3 times of BPPV coding showed an increased incidence of injuries with adjusted HRs of 2.04 (95% CI, 1.90–2.28, *p* < 0.001), 2.47 (95% CI, 2.33–2.71, *p* < 0.001), and 2.63 (95% CI, 2.49–2.88, *p* < 0.001), respectively (Supplementary Table S7).

### 3.3. Different Subtypes of Injury

Supplementary Table S8 shows the differences in subgroups of injuries between patients with and without BPPV. Table 2 shows the results of the Cox regression analyses of injury types in the BPPV cohort. Compared with the non-BPPV cohort, the BPPV cohort exhibited a significantly higher risk of subsequent unintentional injuries (aHR, 2.86 [95% CI, 2.70–3.13]; *p* < 0.001) and intentional injuries (aHR, 1.10 [95% CI, 1.04–1.21]; *p* = 0.012). Notably, among different subtypes of unintentional injuries, patients with BPPVs were at higher risk of falls (aHR, 3.44 [95% CI, 3.25–3.76]) and traffic injuries (aHR, 3.16 [95% CI, 2.98–3.45]) than other subtypes of unintentional injuries. Similarly, the patients with BPPV exhibited a higher risk of injuries with ISSs < 16 (aHR, 2.66 [95% CI, 2.52–2.91]; *p* < 0.001) and ISSs ≥ 16 (aHR, 2.18 [95% CI, 2.06–2.38]; *p* < 0.001) when compared with the controls.

### 3.4. Cumulative Risk of Injuries after Receiving Treatment

Figure 2B presents the results of Kaplan–Meier analysis and the log-rank test for cumulative risk of all-cause injuries stratified by CRT treatment and medication use. Table 3 shows the number of patients who underwent treatment and the effect of various treatment modalities on the risk of injuries in the BPPV cohort. Compared with BPPV patients who did not receive CRT, those who underwent CRT showed a nonsignificantly decreased risk of injury (aHR: 0.78; 95% CI, 0.37–1.29, *p* = 0.67). The patients with BPPV treated with medication (aHR: 0.87, 95% CI: 0.40–1.40, *p* = 0.60), CRT and medication (aHR: 0.68, 95% CI: 0.30–1.14, *p* = 0.73), surgeries (aHR: 0.78, 95% CI: 0.37–1.29, *p* = 0.67), and labyrinthotomy (aHR: 0.64, 95% CI: 0.29–1.12, *p* = 0.74) demonstrated a nonsignificantly reduced risk of injury compared with the patients who did not receive CRT, medication, or any treatment, respectively.

## 4. Discussion

Patients newly diagnosed with BPPV were found to have a higher likelihood of experiencing both unintentional and intentional injuries compared to individuals without BPPV, matched by propensity score. This pattern was consistently seen across various subcategories of unintentional injuries, particularly in cases of falls and traffic accidents. The BPPV group faced greater risks of both minor and major trauma than the non-BPPV group. Additionally, while treatment with CRT or medication might not significantly reduce the injury risk, it implies that physicians should not only focus on managing symptoms and preventing vertigo recurrence but also on advising BPPV patients on injury prevention strategies.

Many studies have reported that PVD is associated with an increased risk of injuries, such as falls [13,19,20,21,22,23,24], traffic accidents [17], and any other injuries [25,26,27]. Kim et al. found that patients visiting emergency departments for acute peripheral vertigo had a higher risk of a new injury within a year [25]. Liao et al. observed that patients with BPPV exhibited a higher risk of fracture than patients without BPPV [26]. Schlick et al. demonstrated that the prevalence rate of recurrent fallers was 30% in bilateral vestibular failure and peripheral neuropathy [24]. Lin et al. found that patients with PVD have a 2-fold higher risk of land transport accidents [17]. By contrast, our findings showed that all subgroups of unintentional injuries except electric current injury and intentional injuries, such as suicide, were associated with the presence of BPPV. An increased risk of intentional injury, specifically suicide, may be because the secondary psychological problems caused by BPPV symptoms affect the daily lives of patients. Other studies have revealed an increased incidence of anxiety and depression in patients with PVD [15,28,29,30,31,32,33,34]. These disorders may predispose an occurrence of intentional injury in long-term follow-up. Therefore, preventive measures and psychological support should be considered in addition to medical treatment for patients with BPPV. Notably, the severity of those injuries might range from minor (ISS < 16) to major trauma (ISS ≥ 16).

Our findings showed that having BPPV was associated with a higher risk of unintentional injuries. Among the subgroups of unintentional injuries, traffic injuries and falls were the two injury types with the highest risk. According to a study by Zhang et al. [13], patients with BPPV have significantly impaired walking stability even when a conservative gait is adopted. Consequently, patients with BPPV may experience an increased incidence of falls [35,36,37]. Furthermore, the incidence of BPPV is higher in older patients [35,36]. The occurrence of vertigo attacks in older patients increases the risk of falls and other unintentional injuries. These factors might contribute to the increased risk of unintentional injury in our study.

Because vertigo attacks in patients with BPPV can occur abruptly in patients’ daily lives, they may have a higher risk of traffic accidents. Lin et al. showed that patients with PVD have a 2-fold higher risk of land transport accidents [17]. Furthermore, this study demonstrated an increased risk of traffic accidents in patients with BPPV. We speculated that head rotations during driving might trigger an abrupt onset of vertigo, which affects mainly horizontal canal BPPV, and causes traffic accidents.

Although studies have shown that CRT, specifically the Epley maneuver [37,38,39,40,41], is an effective and costless treatment for BPPV, our findings showed that CRT had a nonsignificant effect on the risk of subsequent injury. We speculated that although posterior canal BPPVs are predominant, the database did not provide information regarding which semicircular canal was involved and which type of CRT was performed on the patients. Lacking this important information might influence the results of the study. Power et al. [42] reported that compared with the results of the Epley maneuver for posterior canal BPPV, Barbecue and Yacovino CRTs are less effective in horizontal and superior canal BPPVs. However, these BPPV types are less common. BPPV involving horizontal and superior canals may partially explain why we observed an insignificant effect in reducing subsequent injuries in the BPPV cohort. Additionally, the reimbursement of the CRT began in 2012; instead of performing office-based CRTs in the clinical setting, some physicians offered paper-based information guidance on the CRT procedures for BPPV patients to practice home-based CRT. Consequently, these home-based CRTs might underestimate the true incidence of CRT procedures; hence, we should interpret the effect of CRT on the subsequent risk of injuries conservatively. By contrast, the treatment effect of medication for patients with BPPV is controversial. A randomized clinical trial showed that medication does not alleviate residual dizziness after successful repositioning maneuvers [43]. In addition, a meta-analysis showed that vestibular suppressants had little effect on symptom resolution at the longest follow-up period in patients with BPPV [44]. An evidence-based review found no evidence for the use of any medication in the routine treatment for BPPV [45], and a randomized controlled trial observed no difference in symptom resolution, length of stay in the emergency department, or patient satisfaction between standard medical care and CRT [46]. In addition, Casani et al. reported that supplementation with polyphenol compound is safe and manageable and can reduce subjective symptoms and improve instability earlier, decreasing the risk of potential complications [47]. This study evaluated the potential effect of long-term (≥3 months) use of multiple anti-vertigo medications on reducing the occurrence of acute vertiginous episodes and, therefore, decreasing the subsequent risk of injury. Unfortunately, we did not perform a subgroup analysis comparing individual medication categories in reducing the likelihood of injury in patients diagnosed with BPPV. Nonetheless, our findings showed that medication and labyrinthotomy did not have a significant effect on the occurrence of injury in patients with BPPV. Therefore, even after receiving CRT, medication, or a combination of both treatments and surgeries [48], physicians should caution patients with BPPV about their increased risk of injury, particularly older patients with BPPV.

This study has several strengths. First, this population-based cohort study established an association between BPPV and all-cause injuries. Second, previous studies had a short follow-up period. Considering BPPV’s recurrence nature and its increasing prevalence with age, our study evaluated the long-term effects of BPPV on the risk of injury, with an average follow-up period of 14.7 years. Third, we calculated the aHRs for various subgroups according to the specific cause of external injury, providing preventive strategies and potential interventions. Our findings indicate that patients with specific PVDs, particularly BPPV, may exhibit a higher risk of numerous unintentional and intentional injuries than patients without PVD during follow-up. Finally, this study evaluates the potential treatment effects of BPPV on the risk of subsequent injury, concluding that CRT or medication may not affect the risk of subsequent injury after a diagnosis of BPPV.

This study has several limitations. First, detailed medical records for BPPV, including its subtypes and treatment modalities, were unavailable in the claims data. However, to enhance the accuracy of our BPPV definition, we included patients with diagnostic codes from at least three visits and limited our sample to those diagnosed by otolaryngologists and neurologists. Second, the NHI database did not record residual confounding factors such as genetic, physical, behavioral, psychological, and other socioenvironmental parameters related to various types of injuries. Third, the findings from this population-based study may not be applicable to countries with different ethnic and cultural backgrounds. Lastly, the retrospective study design restricted our ability to establish a causal relationship between BPPV and injuries. Additional prospective clinical trials are necessary to clarify the causal relationship between BPPV and subsequent injuries and to assess the impact of treatment on injury risk in BPPV patients.

## 5. Conclusions

Patients with BPPV had a 2.63-fold increased risk of subsequent unintentional and intentional injuries. Patients with BPPV treated with CRT, medication, both, and surgery showed a nonsignificantly reduced risk of subsequent injuries compared with those who did not receive treatments. Our findings indicate that otolaryngologists and neurologists should advocate measures to prevent subsequent injuries in patients with BPPV.

## Figures and Tables

**Table 1:** Baseline characteristics of the study population.

BPPV	Total		With BPPV		Without BPPV		*p*-Value *
Variables	n	%	n	%	n	%	
**Total**	253,375		50,675	20.00	202,700	80.00	
**Sex**							0.999
Men	105,620	41.69	21,124	41.69	84,496	41.69	
Women	147,755	58.31	29,551	58.31	118,204	58.31	
**Age (years)** **(Mean ± SD)**	60.11 ± 15.84		60.01 ± 15.64		60.13 ± 15.89		0.127
**Age group (years)**							0.999
18–29	11,450	4.52	2290	4.52	9160	4.52	
30–39	18,320	7.23	3664	7.23	14,656	7.23	
40–49	32,560	12.85	6512	12.85	26,048	12.85	
50–59	47,945	18.92	9589	18.92	38,356	18.92	
≥60	143,100	56.48	28,620	56.48	114,480	56.48	
**Insured premium** ** **(TWD)**							**<0.001**
<15,840	195,032	76.97	39,301	77.56	155,731	76.83	
15,841–25,000	41,648	16.44	8446	16.67	33,202	16.38	
>25,001	16,695	6.59	2928	5.78	13,767	6.79	
**HTN**							0.580
Without	163,996	64.72	32,746	64.62	131,250	64.75	
With	89,379	35.28	17,929	35.38	71,450	35.25	
**DM**							0.618
Without	204,625	80.76	40,887	80.68	163,738	80.78	
With	48,750	19.24	9788	19.32	38,962	19.22	
**Depression**							0.135
Without	248,689	98.15	49,697	98.07	198,992	98.17	
With	4686	1.85	978	1.93	3708	1.83	
**CHF**							0.501
Without	247,724	97.77	49,525	97.73	198,199	97.78	
With	5651	2.23	1150	2.27	4501	2.22	
**CVA**							0.929
Without	218,559	86.26	43,718	86.27	174,841	86.26	
With	34,816	13.74	6957	13.73	27,859	13.74	
**COPD**							0.932
Without	235,228	92.84	47,050	92.85	188,178	92.84	
With	18,147	7.16	3625	7.15	14,522	7.16	
**Liver cirrhosis**							0.990
Without	238,248	94.03	47,649	94.03	190,599	94.03	
With	15,127	5.97	3026	5.97	12,101	5.97	
**Alcoholism**							0.735
Without	252,496	99.65	50,495	99.64	202,001	99.66	
With	879	0.35	180	0.36	699	0.34	
**CKD**							0.350
Without	246,764	97.39	49,323	97.33	197,441	97.41	
With	6611	2.61	1352	2.67	5259	2.59	
**Migraine**							0.716
Without	250,324	98.80	50,057	98.78	200,267	98.80	
With	3051	1.20	618	1.22	2433	1.20	
**Osteoporosis**							0.569
Without	252,425	99.63	50,478	99.61	201,947	99.63	
With	950	0.37	197	0.39	753	0.37	
**Hyperlipidemia**							0.893
Without	233,901	92.31	46,773	92.30	187,128	92.32	
With	19,474	7.69	3902	7.70	15,572	7.68	
**Autoimmune disease**						0.844	
Without	252,361	99.60	50,475	99.61	201,886	99.60	
With	1014	0.40	200	0.39	814	0.40	
**Season** ***							0.999
Spring (March–May)	64,865	25.60	12,973	25.60	51,892	25.60	
Summer (June–August)	64,755	25.56	12,951	25.56	51,804	25.56	
Autumn (September–November)	64,930	25.63	12,986	25.63	51,944	25.63	
Winter (December–February)	58,825	23.22	11,765	23.22	47,060	23.22	
**Location** (place of residence )							**<0.001**
Northern Taiwan	64,069	25.29	12,020	23.72	52,049	25.68	
Central Taiwan	68,649	27.09	22,048	43.51	46,601	22.99	
Southern Taiwan	60,783	23.99	12,486	24.64	48,297	23.83	
Eastern Taiwan	25,099	9.91	3815	7.53	21,284	10.50	
Outlying islands	34,775	13.72	306	0.60	34,469	17.00	
**Urbanization level** ****							**<0.001**
1 (The highest)	67,298	26.56	10,286	20.30	57,012	28.13	
2	92,163	36.37	22,456	44.31	69,707	34.39	
3	41,683	16.45	7382	14.57	34,301	16.92	
4 (The lowest)	52,231	20.61	10,551	20.82	41,680	20.56	
**Level of care**							**<0.001**
Hospital center	81,233	32.06	10,898	21.51	70,335	34.70	
Regional hospital	94,049	37.12	21,803	43.03	72,246	35.64	
District hospital	78,093	30.82	17,974	35.47	60,119	29.66	

n: number. TWD: New Taiwan Dollar. * *p*-value: Chi-square/Fisher’s exact test on categorical variables and *t*-test on continuous variables; significant *p*-value was <0.05. ** Insured premium levels were used to reflect the insured individual’s socioeconomic status. *** Refer to the season when the injury occurred in both cohorts, or the last visit date when the participants did not experience any injury event. **** The urbanization level was defined by population and certain indicators of the city’s level of development. HTN: hypertension, DM: diabetes mellitus, CHF: chronic heart failure, CVA: cerebrovascular accident, COPD: chronic obstructive pulmonary disease, CKD: chronic kidney disease. There was a statistically significant difference between the experiment and control groups (*p* < 0.05).

**Table 2:** Risk of injury types between patients with and without BPPV by using Cox regression and Bonferroni correction for multiple comparisons.

BPPV	With BPPV			Without BPPV (Reference)			With vs. Without (Reference)		
Injury Subgroup *	Events	PYs	Rate (per 10^5^ PYs)	Events	PYs	Rate (per 10^5^ PYs)	Adjusted HR	95% CI	*p*-Value
Overall	13,215	709,956.75	1861.38	34,421	3,014,434.81	1141.87	2.63	2.49–2.88	<0.001
Unintentional injury	9346	709,956.75	1316.42	22,412	3,014,434.81	743.49	2.86	2.70–3.13	<0.001
Traffic injuries	2308	709,956.75	325.09	5012	3,014,434.81	166.27	3.16	2.98–3.45	<0.001
Poisoning	201	709,956.75	28.31	704	3,014,434.81	23.35	1.96	1.85–2.14	<0.001
Falls	4235	709,956.75	596.52	8432	3,014,434.81	279.72	3.44	3.25–3.76	<0.001
Burns and fires	18	709,956.75	2.54	65	3,014,434.81	2.16	1.90	1.79–2.08	<0.001
Suffocation	40	709,956.75	5.63	142	3,014,434.81	4.71	1.93	1.82–2.11	<0.001
Crushing/cutting/piercing	33	709,956.75	4.65	115	3,014,434.81	3.81	1.97	1.86–2.15	<0.001
Injury caused by animal	18	709,956.75	2.54	79	3,014,434.81	2.62	1.56	1.48–1.71	<0.001
Other unintentional injuries	2493	709,956.75	351.15	7863	3,014,434.81	260.84	2.17	2.05–2.38	<0.001
Intentional injury	29	709,956.75	4.08	180	3,014,434.81	5.97	1.10	1.04–1.21	0.012
Suicide	17	709,956.75	2.39	101	3,014,434.81	3.35	1.15	1.09–1.26	0.003
Homicide/abuse	12	709,956.75	1.69	79	3,014,434.81	2.62	1.04	0.98–1.14	0.086
Intention unknown	4	709,956.75	0.56	18	3,014,434.81	0.60	1.52	1.44–1.67	<0.001
Without E-Code	3836	709,956.75	540.31	11,811	3,014,434.81	391.81	2.23	2.10–2.43	<0.001
ISS ** < 16	12,500	709,956.75	1760.67	32,171	3,014,434.81	1067.23	2.66	2.52–2.91	<0.001
ISS ≥ 16 (Major trauma)	715	709,956.75	100.71	2250	3,014,434.81	74.64	2.18	2.06–2.38	<0.001

* Injury subgroup: derived from the registered *ICD* E-codes of the patients. The E-codes described the events, circumstances, and conditions that cause the effects of an injury coded in the data and specified the external causes for supplemental categorization of the injuries. PYs: person-years; Adjusted HR: adjusted hazard ratio, adjusted for the variables listed in Table 1; CI: confidence interval ** ISS: Injury Severity Score There was a statistically significant difference between the experiment and control groups (significant *p*-value was <0.05).

**Table 3:** Effects of canalith repositioning therapy, medications, and surgeries on the risk of injury between the BPPV and non-BPPV cohorts by using Cox regression and Bonferroni correction for multiple comparisons.

BPPV Subgroup	Populations	Events	PYs	Rate (per 10^5^ PYs)	Adjusted HR	95% CI	*p*-Value *	Adjusted HR	95% CI	*p*-Value *
Without BPPV	202,700	34,421	3,014,434.81	1141.87	Reference					
With BPPV	50,675	13,215	709,956.75	1861.38	2.63	2.49–2.88	<0.001			
BPPV with vs. without CRT										
Without CRT	38,192	10,531	535,070.36	1968.15	2.78	2.63–3.04	<0.001	Reference		
With CRT	12,483	2684	174,886.39	1534.71	2.17	2.05–2.37	<0.001	0.78	0.37–1.29	0.672
BPPV with vs. without medications										
Without medications	26,495	7347	371,195.53	1979.28	2.80	2.64–3.06	<0.001	Reference		
With medications	24,180	5868	338,761.22	1732.19	2.45	2.31–2.68	<0.001	0.87	0.40–1.40	0.597
BPPV with vs. without CRT or medications										
Without CRT or medications	19,378	5740	271,495.38	2114.22	2.99	2.82–3.27	<0.001	Reference		
With CRT only	7117	1607	99,700.15	1611.83	2.28	2.15–2.49	<0.001	0.76	0.35–1.25	0.689
With medications only	18,814	4791	263,574.98	1817.70	2.57	2.43–2.81	<0.001	0.86	0.40–1.39	0.624
With CRT and medications	5366	1077	75,186.24	1432.44	2.03	1.91–2.22	<0.001	0.68	0.30–1.14	0.732
BPPV with vs. without surgeries										
Without ear surgeries	34,974	9804	490,092.50	2000.44	2.83	2.68–3.09	<0.001	Reference		
With ear surgeries	15,701	3411	219,864.25	1551.41	2.19	2.07–2.40	<0.001	0.78	0.37–1.29	0.674
Without labyrinthotomy	50,605	13,203	708,967.29	1862.29	2.63	2.49–2.88	<0.001	Reference		
With labyrinthotomy	70	12	989.46	1212.78	1.71	1.61–1.87	<0.001	0.64	0.29–1.12	0.741

BPPV: benign paroxysmal positional vertigo; PYs: Person-years; Adjusted HR: adjusted Hazard ratio, adjusted for the variables listed in Table 1; CI: confidence interval; CRT: canalith repositioning therapy * significant *p*-value was <0.05.

## Data Availability

The data presented in this study are available upon request from the corresponding author (J.-W.C.). The data are not publicly available due to privacy and ethical restrictions.

## Supplementary Materials

The following supporting information can be downloaded at: https://www.mdpi.com/article/10.3390/jcm13154561/s1, Table S1: The diagnostic codes (ICD-9-CM and ICD-10-CM codes) for the inclusion and exclusion variables and the NHI codes, ATC codes for medications; Table S2: Comparison of years of follow-up and years to injury in the BPPV and non-BPPV cohorts; Table S3: Characteristics of the study population at the end of follow-up; Table S4: Risk factors for the occurrence of injuries by using Cox regression analysis; Table S5: Factors for the occurrence of injuries stratified by variables listed in Table 1 by using Cox regression analysis; Table S6: Sensitivity analyses using different time points and definitions of the comorbidities; Table S7: Differences in incidence of injuries in patients with ≥1, ≥2, and ≥3 times of BPPV diagnosis; Table S8: Differences in subtypes of injuries between patients with and without BPPV.

## References

[1] Libonati G.A., Martellucci S., Castellucci A., Malara P. (2022). Minimum Stimulus Strategy: A step-by-step diagnostic approach to BPPV. J. Neurol. Sci..

[2] Imai T., Takeda N., Ikezono T., Shigeno K., Asai M., Watanabe Y., Suzuki M. (2017). Classification, diagnostic criteria and management of benign paroxysmal positional vertigo. Auris Nasus Larynx.

[3] Molnár A., Maihoub S., Tamás L., Szirmai Á. (2022). A possible objective test to detect benign paroxysmal positional vertigo. The role of the caloric and video-head impulse tests in the diagnosis. J. Otol..

[4] Imai T., Inohara H. (2022). Benign paroxysmal positional vertigo. Auris Nasus Larynx.

[5] Abdulrahim R., Bhandary B.S.K., Rajeshwary A., Goutham M.K., Bhat V., Saldanha M. (2022). The Role of Video Head Impulse Test (Vhit) in Diagnosing Benign Paroxysmal Positional Vertigo (BPPV). Indian J. Otolaryngol. Head Neck Surg..

[6] von Brevern M., Radtke A., Lezius F., Feldmann M., Ziese T., Lempert T., Neuhauser H. (2007). Epidemiology of benign paroxysmal positional vertigo: A population based study. J. Neurol. Neurosurg. Psychiatry.

[7] Kim J.-S., Zee D.S. (2014). Clinical practice. Benign paroxysmal positional vertigo. N. Engl. J. Med..

[8] Parnes L.S., Agrawal S.K., Atlas J. (2003). Diagnosis and management of benign paroxysmal positional vertigo (BPPV). CMAJ.

[9] Walter J., Azeredo W.J., Greene J.S., Andera L. (2021). Prevalence of “Reversal Nystagmus” in Benign Paroxysmal Positional Vertigo. J. Am. Acad. Audiol..

[10] Güneri E.A., Hancı S., Olgun Y., Durankaya S.M. (2022). 3D Model to Understand the Diagnosis and Treatment of Horizontal Canal BPPV. Turk Arch. Otorhinolaryngol..

[11] Kim M.J., Rhim G.I. (2022). Relationship between orthostatic hypotension and recurrence of benign paroxysmal positional vertigo. Sci. Rep..

[12] Lim Y.-H., Kang K., Lee H.-W., Kim J.-S., Kim S.-H. (2021). Gait in Benign Paroxysmal Positional Vertigo. Front. Neurol..

[13] Zhang Y., Wang H., Yao Y., Liu J., Sun X., Gu D. (2021). Walking stability in patients with benign paroxysmal positional vertigo: An objective assessment using wearable accelerometers and machine learning. J. Neuroeng. Rehabil..

[14] Pauwels S., Casters L., Lemkens N., Lemmens W., Meijer K., Meyns P., van de Berg R., Spildooren J. (2023). Gait and Falls in Benign Paroxysmal Positional Vertigo: A Systematic Review and Meta-analysis. J. Neurol. Phys. Ther..

[15] Makarov S.A., Guseva A.L., Dyukova G.M., Golubev V.L., Danilov A.B. (2020). Clinical and psychological features in patients with incident and recurrent cases of benign paroxysmal positional vertigo. Vestn. Otorinolaringol..

[16] Strupp M., Mandalà M., López-Escámez J.A. (2019). Peripheral vestibular disorders: An update. Curr. Opin. Neurol..

[17] Lin H.-C., Xirasagar S., Wang C.-H., Cheng Y.-F., Liu T.-C., Yang T.-H. (2021). A Nationwide Population-Based Study on the Association between Land Transport Accident and Peripheral Vestibular Disorders. Int. J. Environ. Res. Public Health.

[18] Bennett D.A., Brayne C., Feigin V.L., Barker-Collo S., Brainin M., Davis D., Gallo V., Jetté N., Karch A., Kurtzke J.F. (2015). Development of the standards of reporting of neurological disorders (STROND) checklist: A guideline for the reporting of incidence and prevalence studies in neuroepidemiology. Eur. J. Epidemiol..

[19] Hawke L.J., Barr C.J., McLoughlin J.V. (2021). The frequency and impact of undiagnosed benign paroxysmal positional vertigo in outpatients with high falls risk. Age Ageing.

[20] Huang R.J., Pieper C.F., Whitson H.E., Garrison D.B., Pavon J.M., Riska K.M. (2022). Evaluating the Association Between Hearing Loss and Falls in Adults With Vestibular Dysfunction or Nonvestibular Dizziness. Ear Hear..

[21] Pierchała K., Lachowska M., Wysocki J., Morawski K., Niemczyk K. (2019). Evaluation of the Sensory Organization Test to differentiate non-fallers from single- and multi-fallers. Adv. Clin. Exp. Med..

[22] Balatsouras D., Koukoutsis G., Fassolis A., Moukos A., Aspris A., Apris A. (2018). Benign paroxysmal positional vertigo in the elderly: Current insights. Clin. Interv. Aging.

[23] Huang R.J., Smith S.L., Brezina L., Riska K.M. (2021). A Comparison of Falls and Dizziness Handicap by Vestibular Diagnosis. Am. J. Audiol..

[24] Schlick C., Schniepp R., Loidl V., Wuehr M., Hesselbarth K., Jahn K. (2016). Falls and fear of falling in vertigo and balance disorders: A controlled cross-sectional study. J. Vestib. Res..

[25] Kim H., Lee S., Kim J. (2020). Risk of injury after emergency department visit for acute peripheral vertigo: A matched-cohort study. Clin. Exp. Emerg. Med..

[26] Liao W.-L., Chang T.-P., Chen H.-J., Kao C.-H. (2015). Benign paroxysmal positional vertigo is associated with an increased risk of fracture: A population-based cohort study. J. Orthop. Sports Phys. Ther..

[27] Wu P., Lin H., Chien W., Chung C., Chen J. (2022). Increased Risk of Injury in Ménière’s Disease and Effects of Treatment: Population-Based Retrospective Cohort Study. Otolaryngol. Head Neck Surg..

[28] Bayat A., Hoseinabadi R., Saki N., Sanayi R. (2020). Disability and Anxiety in Vestibular Diseases: A Cross-Sectional Study. Cureus.

[29] Kahraman S.S., Arli C., Copoglu U.S., Kokacya M.H., Colak S. (2017). The evaluation of anxiety and panic agarophobia scores in patients with benign paroxysmal positional vertigo on initial presentation and at the follow-up visit. Acta Oto-Laryngol..

[30] Lahiji M.R., Akbarpour M., Soleimani R., Asli R.H., Leyli E.K., Saberi A., Akbari M., Ramezani H., Nemati S. (2022). Prevalence of anxiety and depression in Meniere’s disease; a comparative analytical study. Am. J. Otolaryngol..

[31] Ozdilek A., Yalinay Dikmen P., Acar E., Ayanoglu Aksoy E., Korkut N. (2019). Determination of Anxiety, Health Anxiety and Somatosensory Amplification Levels in Individuals with Benign Paroxysmal Positional Vertigo. J. Int. Adv. Otol..

[32] Wang L., Zhou H.F., Wang M.X., Zhang J., Su J., Guo Y. (2016). Comparison of anxiety and depression state among patients with different type of benign paroxysmal positional vertigo. J. Clin. Otorhinolaryngol. Head Neck Surg..

[33] Mutlu B., Topcu M.T. (2022). Investigation of the Relationship between Vestibular Disorders and Sleep Disturbance. Int. Arch. Otorhinolaryngol..

[34] Molnar A., Maihoub S., Mavrogeni P., Tamas L., Szirmai A. (2022). Depression scores and quality of life of vertiginous patients, suffering from different vestibular disorders. Eur. Arch. Otorhinolaryngol..

[35] Fujimoto C., Kawahara T., Kinoshita M., Kikkawa Y.S., Sugasawa K., Yagi M., Yamasoba T., Iwasaki S., Murofushi T. (2018). Aging Is a Risk Factor for Utricular Dysfunction in Idiopathic Benign Paroxysmal Positional Vertigo. Front. Neurol..

[36] Liu D.-H., Kuo C.-H., Wang C.-T., Chiu C.-C., Chen T.-J., Hwang D.-K., Kao C.-L. (2017). Age-Related Increases in Benign Paroxysmal Positional Vertigo Are Reversed in Women Taking Estrogen Replacement Therapy: A Population-Based Study in Taiwan. Front. Aging Neurosci..

[37] Hilton M.P., Pinder D.K. (2014). The Epley (canalith repositioning) manoeuvre for benign paroxysmal positional vertigo. Cochrane Database Syst. Rev..

[38] Woodworth B.A., Gillespie M.B., Lambert P.R. (2004). The canalith repositioning procedure for benign positional vertigo: A meta-analysis. Laryngoscope.

[39] Gold D.R., Morris L., Kheradmand A., Schubert M.C. (2014). Repositioning maneuvers for benign paroxysmal positional vertigo. Curr. Treat. Options Neurol..

[40] Brown M.D. (2011). Evidence-based emergency medicine. Is the canalith repositioning maneuver effective in the acute management of benign positional vertigo?. Ann. Emerg. Med..

[41] Helminski J.O., Zee D.S., Janssen I., Hain T.C. (2010). Effectiveness of particle repositioning maneuvers in the treatment of benign paroxysmal positional vertigo: A systematic review. Phys. Ther..

[42] Power L., Murray K., Szmulewicz D.J. (2020). Characteristics of assessment and treatment in Benign Paroxysmal Positional Vertigo (BPPV). J. Vestib. Res..

[43] Acar B., Karasen R.M., Buran Y. (2015). Efficacy of medical therapy in the prevention of residual dizziness after successful repositioning maneuvers for Benign Paroxysmal Positional Vertigo (BPPV). B-ENT.

[44] Sharif S., Khoujah D., Greer A., Naples J.G., Upadhye S., Edlow J.A. (2022). Vestibular suppressants for benign paroxysmal positional vertigo: A systematic review and meta-analysis of randomized controlled trials. Acad. Emerg. Med..

[45] Fife T.D., Iverson D.J., Lempert T., Furman J.M., Baloh R.W., Tusa R.J., Hain T.C., Herdman S., Morrow M.J., Gronseth G.S. (2008). Practice parameter: Therapies for benign paroxysmal positional vertigo (an evidence-based review): Report of the Quality Standards Subcommittee of the American Academy of Neurology. Neurology.

[46] Sacco R.R., Burmeister D.B., Rupp V.A., Greenberg M.R. (2014). Management of benign paroxysmal positional vertigo: A randomized controlled trial. J. Emerg. Med..

[47] Casani A.P., Navari E., Albera R., Agus G., Libonati G.A., Chiarella G., Lombardo N., Marcelli V., Ralli G., di Santillo L.S. (2019). Approach to residual dizziness after successfully treated benign paroxysmal positional vertigo: Effect of a polyphenol compound supplementation. Clin. Pharmacol. Adv. Appl..

[48] Corvera Behar G., Garcia de la Cruz M.A. (2017). Surgical Treatment for Recurrent Benign Paroxysmal Positional Vertigo. Int. Arch. Otorhinolaryngol..

