# Supplementary material for: Incidence of Subsequent Injuries Associated with a New Diagnosis of Benign Paroxysmal Positional Vertigo and Effects of Treatment: A Nationwide Cohort Study
Source: J Clin Med. 2024 Aug 5;13(15):4561. doi: 10.3390/jcm13154561 (PMC11312852; doi:10.3390/jcm13154561)
Supplement: Supplementary file 1 [file jcm-13-04561-s001.zip › jcm-3063930-supplementary.pdf]

Supplemental Material

(Manuscript Title: Incidence of Injuries Associated With a New Diagnosis of Benign Paroxysmal Positional Vertigo and Effects of Treatment: A Nationwide Cohort Study)

Contents

Supplementary Table S1. The diagnostic codes (*ICD-9-CM* and *ICD-10-CM* codes) for the inclusion and exclusion variables and the NHI codes, ATC codes for medications.
Supplementary Table S2. Comparison of years of follow-up and years to injury in the BPPV and non-BPPV cohorts.
Supplementary Table S3. Characteristics of the study population at the end of follow-up.
Supplementary Table S4. Risk factors for the occurrence of injuries by using Cox regression analysis.
Supplementary Table S5. Factors for the occurrence of injuries stratified by variables listed in Table 1 by using Cox regression analysis.
Supplementary Table S6. Sensitivity analyses using different time points and definitions of the comorbidities.
Supplementary Table S7. Differences in incidence of injuries in patients with ≥ 1, ≥ 2, and ≥ 3 times of BPPV diagnosis
Supplementary Table S8. Differences in subtypes of injuries between patients with and without BPPV.

Supplementary Table S1. The diagnostic codes (*ICD-9-CM* and *ICD-10-CM* codes) for the inclusion and exclusion variables and the NHI codes, ATC codes for medications.

	Abbreviations	*ICD-9-CM* / *ICD-10*-CM / NHI Code / ATC Code / Definition
**Study population:**<br>Benign paroxysmal positional vertigo	BPPV	386.11; H81.11-H81.13; Diagnosis by otolaryngologist or neurologist and medical visits ≧ 3; The time interval from the first diagnosis to the third episode in the BPPV cohort was 30.24 ± 24.36 days, and the median was 21 days.
Canalith repositioning therapy	CRT	54044B
Medications		≧ 3 months
Antivertigo preparations		N07C, N07CA
Antiemetics and antinauseants		A04A
Peripheral vasodilators		C04AX
Antimigraine preparations		N02C
Antipsychotics		N05A
Antihistamines for systemic use		R06A
Ear surgeries		Any of the listed below
Exploratory tympanotomy		84016B
Tympanoplasty without mastoidectomy		84018B
Tympanoplasty with mastoidectomy		84019B
Ossiculoplasty		84020B
Mastoidectomy, simple		84021B
Mastoidectomy, modified		84022B
Stapedectomy with prosthesis		84025B
Stapes mobilization		84026B
Transtympanic transmastoid labyrinthectomy		84028B

Supplementary Table S1. continued.

	Abbreviations	*ICD-9-CM* / *ICD-10*-CM / NHI Code / ATC Code / Definition
Endolymphatic sac decompression		84029B
Labyrinthotomy		84030B
Labyrinthectomy		84031B
Translabyrinthine acoustic neuroma excision		84032B
Fenestration of semicircular canals		84036B
Cochlear implantation		84038B
**Excluding:**		
Epidemic vertigo		078.81; A88.1
Benign neoplasm of cranial nerves		225.1; D33.3
Vertiginous syndromes and other disorders of vestibular system		386 excluding 386.11; H81.1-H81.3 excluding H81.11-H81.13; including common vestibular disorders such as Meniere's disease, vestibular neuritis, labyrinthitis, and other unspecified peripheral vertigo
Sudden hearing loss		388.2; H91.2
Dizziness and giddiness		780.4; R42
Cervicogenic dizziness		780.4 and 723.1; M53.0
Postconcussion syndrome		310.2; F07.81
Traumatic head injury	TBI	800-804, 850-854, 870-873, 907.0-907.1, 959, V15.52; S01-S02, S06, T90
Epilepsy		345; G40.A01, G40.A09
Infantile cerebral palsy	ICP	343; G80.1

Supplementary Table S1. continued.

	Abbreviations	***ICD-9-CM* / *ICD-10*-CM / NHI Code / ATC Code / Definition**
**Events:** Injury		800-999; V01-Y89
Unintentional injury		E800-E949; V01-X59, Y40-Y69, Y83-Y86
Traffic injuries		E800-E849; V01-V99
Poisoning		E850-E869; X40-X49
Falls		E880-E888; W00-W19
Burns and fires		E890-E899; X00-X09
Drowning		E910; W65-W74
Suffocation		E911-E915; W75-W84
Crushing / cutting / piercing		E916-E920; W20-W31
Excessive heat		E900; W92
Injury caused by animal		E906; W50-W64
Electric current injury		E925; W85-W87
Other unintentional injuries		E870-E879, E901-E905, E907-E909, E921-E924, E926-E949; W35-W49, W88-W91, W93-W94, X10-X19, X30-X39, X50-X57, Y40-Y69, Y83-Y84
Intentional injury		E950-E979, E990-E999; X60-Y09, Y35-Y36, Y87.0-Y87.1, Y89.0-Y89.1
Suicide		E950-E959; X60-X84, Y87.0
Homicide / abuse		E960-E969; X85-Y09, Y87.1
Intention unknown		E980-E989; Y10-Y34, Y87.2, Y89.9
Injury severity score	ISS	ISS ≥16: Major trauma
**Comorbidities:**		
Hypertension	HTN	401-405; I10-I15
Diabetes mellitus	DM	250; E10-E14
Depression		296.2-296.3, 296.82, 300.4, 311; F32-F33, F34.1

Supplementary Table S1. continued.

	Abbreviations	*ICD-9-CM* / *ICD-10*-CM / NHI Code / ATC Code / Definition
Congestive heart failure	CHF	428; I50
Cerebrovascular accident	CVA	430-438; I60-I69
Chronic obstructive pulmonary disease	COPD	490-496; J40-J47
Liver disease		571; K70-K77excluding K70.9
Alcoholism		291, 303, 571.3; F10, K70.9
Chronic kidney disease	CKD	585-586; N18-N19
Gout		274; M10
Migraine		346; G43
Osteoporosis		733.0; M81
Hyperlipidemia		272.0-272.4; E78.4-E78.5, E78.8-E78.9
Autoimmune disease	AID	710; M32-M35
**Other Medications:**		≧3 months
Anxiolytics		N05B
Labyrinthine sedatives		N05C
Anti-depressants		N06A
Polypharmacy		≧5 types of medication

Supplementary Table S2. Comparison of years of follow-up and years to injury in the BPPV and non-BPPV cohorts.

	Years of follow-up				Years to injury			
**BPPV subgroup**	**Minimum**	**Median**	**Maximum**	**Mean ± SD**	**Minimum**	**Median**	**Maximum**	**Mean ± SD**
With BPPV	0.01	9.35	17.95	14.02 ± 11.98	0.01	6.22	17.52	8.18 ± 5.04
Without BPPV	0.01	9.97	17.97	14.86 ± 12.21	0.01	7.02	17.71	8.89 ± 5.57
Overall	0.01	9.86	17.97	14.69 ± 12.17	0.01	6.97	17.71	8.75 ± 5.48

Supplementary Table S3. Characteristics of the study population at the end of follow-up.

BPPV	Total		With BPPV		Without BPPV		*p-value**
**Variables**	**n**	**%**	**n**	**%**	**n**	**%**	
**Total**	253,375		50,675	20.00	202,700	80.00	
**Injury**							< 0.001
Without	205,739	81.20	37,460	73.92	168,279	83.02	
With	47,636	18.80	13,215	26.08	34,421	16.98	
**Sex**							0.999
Male	105,620	41.69	21,124	41.69	84,496	41.69	
Female	147,755	58.31	29,551	58.31	118,204	58.31	
**Age (years) (Mean ± SD)**	74.80 ± 16.90		74.03 ± 16.38		74.99 ± 17.02		< 0.001
**Age group (years)**							< 0.001
18 - 29	9,599	3.79	2,077	4.10	7,522	3.71	
30 - 39	17,097	6.75	3,765	7.43	13,332	6.58	
40 - 49	25,937	10.24	5,823	11.49	20,114	9.92	
50 - 59	38,792	15.31	8,586	16.94	30,206	14.90	
≧ 60	161,950	63.92	30,424	60.04	131,526	64.89	
**Insured premium** (NT$)**							< 0.001
< 15,840	195,032	76.97	39,301	77.56	155,731	76.83	
15,841 - 25,000	41,648	16.44	8,446	16.67	33,202	16.38	
> 25,001	16,695	6.59	2,928	5.78	13,767	6.79	
**HTN**							< 0.001
Without	196,702	77.63	35,703	70.45	160,999	79.43	
With	56,673	22.37	14,972	29.55	41,701	20.57	

Supplementary Table S3. Continued.

BPPV	Total		With BPPV		Without BPPV		*p-value**
Variables	n	%	n	%	n	%	
**DM**							< 0.001
Without	206,545	81.52	40,989	80.89	165,556	81.68	
With	46,830	18.48	9,686	19.11	37,144	18.32	
**Depression**							< 0.001
Without	250,521	98.87	49,640	97.96	200,881	99.10	
With	2,854	1.13	1,035	2.04	1,819	0.90	
**CHF**							< 0.001
Without	245,583	96.92	48,779	96.26	196,804	97.09	
With	7,792	3.08	1,896	3.74	5,896	2.91	
**CVA**							< 0.001
Without	227,381	89.74	43,653	86.14	183,728	90.64	
With	25,994	10.26	7,022	13.86	18,972	9.36	
**COPD**							0.066
Without	234,352	92.49	46,773	92.30	187,579	92.54	
With	19,023	7.51	3,902	7.70	15,121	7.46	
**Liver cirrhosis**							< 0.001
Without	239,916	94.69	47,361	93.46	192,555	95.00	
With	13,459	5.31	3,314	6.54	10,145	5.00	
**Alcoholism**							0.450
Without	252,486	99.65	50,488	99.63	201,998	99.65	
With	889	0.35	187	0.37	702	0.35	

Supplementary Table S3. Continued.

BPPV	Total		With BPPV		Without BPPV		*p-value**
Variables	n	%	n	%	n	%	
**CKD**							< 0.001
Without	244,475	96.49	48,476	95.66	195,999	96.69	
With	8,900	3.51	2,199	4.34	6,701	3.31	
**Migraine**							< 0.001
Without	251,400	99.22	49,973	98.61	201,427	99.37	
With	1,975	0.78	702	1.39	1,273	0.63	
**Osteoporosis**							0.188
Without	250,812	98.99	50,136	98.94	200,676	99.00	
With	2,563	1.01	539	1.06	2,024	1.00	
**Hyperlipidemia**							< 0.001
Without	241,569	95.34	47,842	94.41	193,727	95.57	
With	11,806	4.66	2,833	5.59	8,973	4.43	
**AID**							0.038
Without	252,316	99.58	50,436	99.53	201,880	99.60	
With	1,059	0.42	239	0.47	820	0.40	
**Season*****							< 0.001
Spring	63,456	25.04	12,086	23.85	51,370	25.34	
Summer	63,863	25.20	12,879	25.41	50,984	25.15	
Autumn	64,166	25.32	13,004	25.66	51,162	25.24	
Winter	61,890	24.43	12,706	25.07	49,184	24.26	

Supplementary Table S3. Continued.

BPPV	Total		With BPPV		Without BPPV		*p-value**
**Variables**	**n**	**%**	**n**	**%**	**n**	**%**	
**Location (place of residence)**							< 0.001
Northern Taiwan	72,676	28.68	12,975	25.60	59,701	29.45	
Central Taiwan	76,962	30.37	21,158	41.75	55,804	27.53	
Southern Taiwan	69,676	27.50	12,541	24.75	57,135	28.19	
Eastern Taiwan	30,115	11.89	3,535	6.98	26,580	13.11	
Outlying islands	3,946	1.56	466	0.92	3,480	1.72	
**Urbanization level********							< 0.001
1 (The highest)	65,607	25.89	12,597	24.86	53,010	26.15	
2	87,464	34.52	19,020	37.53	68,444	33.77	
3	40,800	16.10	5,786	11.42	35,014	17.27	
4 (The lowest)	59,504	23.48	13,272	26.19	46,232	22.81	
**Level of care**							< 0.001
Hospital center	82,754	32.66	11,656	23.00	71,098	35.08	
Regional hospital	99,765	39.37	22,081	43.57	77,684	38.32	
District hospital	70,856	27.96	16,938	33.42	53,918	26.60	

*P: Chi-square/Fisher's exact test on categorical variables and t-test on continuous variables

**Insured premium levels were used to reflect the insured individual's socioeconomic status.

***Refer to the season when the injury occurred in both cohorts, or the last visit date when the participants did not experience any injury event.

****The urbanization level was defined by population and certain indicators of the city's level of development.

Supplementary Table S4. Risk factors for the occurrence of injuries by using Cox regression analysis.

Variables	Crude HR	95% CI	95% CI	*p-value**	Adjusted HR	95% CI	95% CI	*p-value**
**BPPV**								
Without	Reference				Reference			
With	2.98	2.56	3.39	< 0.001	2.63	2.49	2.88	< 0.001
**Gender**								
Male	1.36	1.16	1.58	< 0.001	1.26	1.08	1.45	0.006
Female	Reference				Reference			
**Age (yrs)**								
18 - 29	Reference				Reference			
30 - 39	0.92	0.45	1.10	0.104	0.97	0.50	1.21	0.136
40 - 49	0.63	0.35	0.98	0.036	0.74	0.40	1.09	0.079
50 - 59	0.52	0.30	0.91	0.004	0.69	0.35	0.97	0.019
≧ 60	0.88	0.43	1.07	0.076	0.93	0.48	1.17	0.118
**Insured premium** (NT$)**								
< 15,840	Reference				Reference			
15,841 - 25,000	0.93	0.60	1.38	0.429	0.90	0.50	1.33	0.496
> 25,001	0.89	0.53	1.31	0.452	0.81	0.48	1.28	0.533
**HTN**								
Without	Reference				Reference			
With	1.99	1.68	2.44	< 0.001	1.87	1.56	2.13	< 0.001
**DM**								
Without	Reference				Reference			
With	1.30	1.19	1.57	< 0.001	1.23	1.10	1.41	0.001

Supplementary Table S4. Continued.

Variables	Crude HR	95% CI	95% CI	*p-value**	Adjusted HR	95% CI	95% CI	*p-value**
**Depression**								
Without	Reference				Reference			
With	1.34	1.09	1.57	0.001	1.30	1.05	1.53	0.022
**CHF**								
Without	Reference				Reference			
With	1.73	1.10	2.40	0.001	1.53	0.94	2.31	0.096
**CVA**								
Without	Reference				Reference			
With	1.97	1.57	2.53	< 0.001	1.72	1.37	2.27	< 0.001
**COPD**								
Without	Reference				Reference			
With	1.45	1.00	1.87	0.048	1.04	0.79	1.37	0.219
**Liver cirrhosis**								
Without	Reference				Reference			
With	2.24	1.77	2.97	< 0.001	1.57	1.33	1.88	< 0.001
**Alcoholism**								
Without	Reference				Reference			
With	1.54	1.20	2.03	< 0.001	1.49	1.10	1.90	< 0.001
**CKD**								
Without	Reference				Reference			
With	1.42	1.09	1.63	0.007	1.40	1.02	1.58	0.030

Supplementary Table S4. Continued.

Variables	Crude HR	95% CI	95% CI	*p-value**	Adjusted HR	95% CI	95% CI	*p-value**
**Migraine**								
Without	Reference				Reference			
With	2.77	1.79	3.90	< 0.001	1.79	1.36	2.17	< 0.001
**Osteoporosis**								
Without	Reference				Reference			
With	2.33	1.86	2.71	< 0.001	2.00	1.56	2.44	< 0.001
**Hyperlipidemia**								
Without	Reference				Reference			
With	1.78	1.58	1.97	< 0.001	1.53	1.33	1.73	<0.001
**AID**								
Without	Reference				Reference			
With	1.31	0.97	1.55	0.097	1.20	0.86	1.47	0.184
**Season***								
Spring	Reference				Reference			
Summer	0.83	0.52	1.07	0.481	0.95	0.63	1.19	0.489
Autumn	0.82	0.51	1.06	0.486	0.95	0.62	1.19	0.477
Winter	0.73	0.43	0.97	0.011	0.81	0.55	1.03	0.401
**Location**					Multicollinearity with urbanization level			
Northern Taiwan	Reference							
Central Taiwan	1.09	0.81	1.33	0.167				
Southern Taiwan	0.99	0.75	1.27	0.252				
Eastern Taiwan	0.92	0.73	1.24	0.259				
Outlying islands	0.80	0.67	1.19	0.342				

Supplementary Table S4. Continued.

Variables	Crude HR	95% CI	95% CI	*p-value**	Adjusted HR	95% CI	95% CI	*p-value**
**Urbanization level********								
1 (The highest)	1.38	1.23	1.50	< 0.001	1.28	1.10	1.39	< 0.001
2	1.20	1.10	1.37	0.002	1.14	1.00	1.30	0.048
3	1.10	1.00	1.24	0.050	1.01	0.93	1.18	0.184
4 (The lowest)	Reference				Reference			
**Level of care**								
Hospital center	2.24	1.90	2.71	< 0.001	1.86	1.67	2.06	< 0.001
Regional hospital	1.77	1.46	2.18	< 0.001	1.57	1.34	1.79	< 0.001
District hospital	Reference				Reference			

*P: Chi-square/Fisher's exact test on categorical variables and t-test on continuous variables

**Insured premium levels were used to reflect the insured individual's socioeconomic status.

***Refer to the season when the injury occurred in both cohorts, or the last visit date when the participants did not experience any injury event.

****The urbanization level was defined by population and certain indicators of the city's level of development.

HR: hazard ratio; CI: confidence interval; Adjusted HR: adjusted variables listed in the table

Supplementary Table S5. Factors for the occurrence of injuries stratified by variables listed in Table 1 by using Cox regression analysis and Bonferroni correction for multiple comparisons.

BPPV	With BPPV			Without BPPV *(Reference)*			With *vs.* Without *(Reference)*			
**Stratified**	**Events**	**PYs**	**Rate (per 10^5 PYs)**	**Events**	**PYs**	**Rate (per 10^5 PYs)**	**Adjusted HR**	**95% CI**	**95% CI**	***p-value****
**Total**	13,215	709,956.75	1,861.38	34,421	3,014,434.81	1,141.87	2.63	2.49	2.88	< 0.001
**Sex**										
Male	5,861	295,974.11	1,980.24	14,241	1,256,572.06	1,133.32	2.82	2.67	3.08	< 0.001
Female	7,354	413,982.64	1,776.40	20,180	1,757,862.75	1,147.98	2.50	2.36	2.73	< 0.001
**Age (yrs)**										
18 - 29	556	29,098.76	1,910.73	1,283	111,862.42	1,146.94	2.69	2.54	2.94	< 0.001
30 - 39	989	52,745.11	1,875.06	2,267	198,265.13	1,143.42	2.65	2.50	2.89	< 0.001
40 - 49	1,513	81,580.26	1,854.62	3,412	299,123.50	1,140.67	2.62	2.48	2.87	< 0.001
50 - 59	2,221	120,289.35	1,846.38	5,120	449,208.81	1,139.78	2.62	2.47	2.86	< 0.001
≧ 60	7,936	426,243.27	1,861.85	22,339	1,955,974.95	1,142.09	2.63	2.49	2.88	< 0.001
**Insured premium** (NT$)**										
< 15,840	10,331	550,670.22	1,876.08	26,483	2,315,979.22	1,143.49	2.65	2.50	2.90	< 0.001
15,841 - 25,000	2,205	118,328.46	1,863.46	5,641	493,760.18	1,142.46	2.63	2.49	2.88	< 0.001
> 25,001	679	40,958.07	1,657.79	2,297	204,695.41	1,122.16	2.38	2.25	2.61	< 0.001
**HTN**										
Without	9,220	500,234.53	1,843.14	27,338	2,394,282.65	1,141.80	2.61	2.46	2.85	< 0.001
With	3,995	209,722.22	1,904.90	7,083	620,152.16	1,142.14	2.69	2.54	2.94	< 0.001
**DM**										
Without	10,604	574,256.49	1,846.56	28,107	2,462,049.80	1,141.61	2.61	2.47	2.86	< 0.001
With	2,611	135,700.26	1,924.09	6,314	552,385.01	1,143.04	2.72	2.57	2.97	< 0.001
**Depression**										
Without	12,880	695,451.39	1,852.03	34,079	2,987,375.33	1,140.77	2.62	2.48	2.87	< 0.001

With	335	14,505.36	2,309.49	342	27,059.48	1,263.88	2.95	2.79	3.23	< 0.001
**CHF**										
Without	12,707	683,393.78	1,859.40	33,415	2,926,750.56	1,141.71	2.63	2.48	2.87	< 0.001
With	508	26,562.97	1,912.44	1,006	87,684.25	1,147.30	2.69	2.54	2.94	< 0.001
**CVA**										
Without	11,351	611,581.74	1,856.01	31,196	2,732,287.84	1,141.75	2.62	2.48	2.87	< 0.001
With	1,864	98,375.01	1,894.79	3,225	282,146.97	1,143.02	2.68	2.53	2.93	< 0.001
**COPD**										
Without	12,182	655,289.55	1,859.03	31,854	2,789,626.46	1,141.87	2.63	2.48	2.87	< 0.001
With	1,033	54,667.20	1,889.62	2,567	224,808.35	1,141.86	2.67	2.52	2.92	< 0.001
**Liver cirrhosis**										
Without	12,312	663,535.65	1,855.51	32,696	2,863,564.59	1,141.79	2.62	2.48	2.87	< 0.001
With	903	46,421.10	1,945.24	1,725	150,870.22	1,143.37	2.75	2.60	3.00	< 0.001
**Alcoholism**										
Without	13,151	707,338.61	1,859.22	34,292	3,003,931.52	1,141.57	2.63	2.48	2.87	< 0.001
With	64	2,618.14	2,444.48	129	10,503.29	1,228.19	3.21	3.04	3.51	< 0.001
**CKD**										
Without	12,594	679,149.63	1,854.38	33,279	2,914,783.32	1,141.73	2.62	2.48	2.87	< 0.001
With	621	30,807.12	2,015.77	1,142	99,651.49	1,145.99	2.84	2.68	3.10	< 0.001
**Migraine**										
Without	12,974	700,191.66	1,852.92	34,191	2,995,487.95	1,141.42	2.62	2.48	2.87	< 0.001
With	241	9,765.09	2,467.98	230	18,946.86	1,213.92	3.28	3.10	3.59	< 0.001
**Osteoporosis**										
Without	13,067	702,405.53	1,860.32	34,076	2,984,337.25	1,141.83	2.63	2.49	2.88	< 0.001
With	148	7,551.22	1,959.95	345	30,097.56	1,146.27	2.76	2.61	3.02	< 0.001
**Hyperlipidemia**										

Without	12,471	670,326.53	1,860.44	32,893	2,880,992.75	1,141.72	2.63	2.49	2.88	< 0.001
With	744	39,630.22	1,877.36	1,528	133,442.06	1,145.07	2.65	2.50	2.89	< 0.001
**AID**										
Without	13,149	706,607.83	1,860.86	34,279	3,002,240.46	1,141.78	2.63	2.49	2.88	< 0.001
With	66	3,348.92	1,970.78	142	12,194.35	1,164.47	2.73	2.58	2.99	< 0.001
**Season*** **										
Spring	3,224	169,324.25	1,904.04	8,795	763,975.22	1,151.22	2.67	2.52	2.92	< 0.001
Summer	3,372	180,434.11	1,868.83	8,701	758,202.11	1,147.58	2.63	2.48	2.87	< 0.001
Autumn	3,391	182,186.05	1,861.28	8,796	768,701.75	1,144.27	2.63	2.48	2.87	< 0.001
Winter	3,228	178,012.34	1,813.36	8,129	723,555.73	1,123.48	2.61	2.46	2.85	< 0.001
**Urbanization level** ****										
1 (The highest)	3,301	176,483.79	1,870.43	9,006	788,335.12	1,142.41	2.64	2.50	2.89	< 0.001
2	4,963	266,470.01	1,862.50	11,625	1,017,852.46	1,142.11	2.63	2.49	2.88	< 0.001
3	1,515	81,608.88	1,856.42	5,945	520,707.29	1,141.72	2.62	2.48	2.87	< 0.001
4 (The lowest)	3,436	185,394.07	1,853.35	7,845	687,539.94	1,141.02	2.62	2.48	2.87	< 0.001
**Level of care**										
Hospital center	3,189	163,302.44	1,952.82	12,098	1,057,327.46	1,144.21	2.76	2.60	3.01	< 0.001
Regional hospital	5,762	309,354.17	1,862.59	13,184	1,155,282.06	1,141.19	2.63	2.49	2.88	< 0.001
District hospital	4,264	237,300.14	1,796.88	9,139	801,825.29	1,139.77	2.55	2.40	2.78	< 0.001

**P*: Chi-square/Fisher's exact test on categorical variables and t-test on continuous variables

**Insured premium levels were used to reflect the insured individual's socioeconomic status.

***Refer to the season when the injury occurred in both cohorts, or the last visit date when the participants did not experience any injury event.

****The urbanization level was defined by population and certain indicators of the city's level of development.

PYs: person-years; Adjusted HR: adjusted hazard ratio, adjusted for variables listed in table 1; CI: confidence interval;

Supplementary Table S6. Sensitivity analyses using different time points and definitions of the comorbidities.

Definition of comorbidities	BPPV	With (n = 50,675)		Without (n = 202,700)			
Baseline	Comorbidities	n	%	n	%	*P*	Risk of injury
	HTN	17,929	35.38	71,450	35.25	0.580	BPPV vs. non-BPPV *(Reference):*
	DM	9,788	19.32	38,962	19.22	0.618	aHR = 2.29 (95% CI = 2.16 – 2.50), *P* < 0.001
	Depression	978	1.93	3,708	1.83	0.135	
	CHF	1,150	2.27	4,501	2.22	0.501	
	CVA	6,957	13.73	27,859	13.74	0.929	
	COPD	3,625	7.15	14,522	7.16	0.932	
	Liver cirrhosis	3,026	5.97	12,101	5.97	0.990	
	Alcoholism	180	0.36	699	0.34	0.735	
	CKD	1,352	2.67	5,259	2.59	0.350	
	Migraine	618	1.22	2,433	1.20	0.716	
	Osteoporosis	197	0.39	753	0.37	0.569	
	Hyperlipidemia	3,902	7.70	15,572	7.68	0.893	
	Autoimmune disease	200	0.39	814	0.40	0.844	
Endpoint	Comorbidities	n	%	n	%	*P*	Risk of injury
	HTN	14,972	29.55	41,701	20.57	< 0.001	BPPV vs. non-BPPV *(Reference):*
	DM	9,686	19.11	37,144	18.32	< 0.001	aHR = 2.63 (95% CI = 2.49 – 2.88), *P* < 0.001
	Depression	1,035	2.04	1,819	0.90	< 0.001	
	CHF	1,896	3.74	5,896	2.91	< 0.001	
	CVA	7,022	13.86	18,972	9.36	< 0.001	
	COPD	3,902	7.70	15,121	7.46	0.066	
	Liver cirrhosis	3,314	6.54	10,145	5.00	< 0.001	
	Alcoholism	187	0.37	702	0.35	0.450	

	CKD	2,199	4.34	6,701	3.31	< 0.001	
	Migraine	702	1.39	1,273	0.63	< 0.001	
	Osteoporosis	539	1.06	2,024	1.00	0.188	
	Hyperlipidemia	2,833	5.59	8,973	4.43	< 0.001	
	Autoimmune disease	239	0.47	820	0.40	0.038	
During study period	Comorbidities	n	%	n	%	*P*	Risk of injury
≧3 visits	HTN	15,042	29.68	60,724	29.96	0.229	BPPV vs. non-BPPV *(Reference):*
≧3 visits	DM	10,243	20.21	41,018	20.24	0.909	aHR = 2.48 (95% CI = 2.34 – 2.71), *P* < 0.001
≧3 visits	Depression	1,215	2.40	2,871	1.42	< 0.001	
≧3 visits	CHF	1,901	3.75	7,396	3.65	0.243	
≧3 visits	CVA	7,145	14.10	27,981	13.80	0.148	
≧3 visits	COPD	4,003	7.90	16,124	7.95	0.681	
≧3 visits	Liver cirrhosis	3,571	7.05	12,497	6.17	< 0.001	
≧3 visits	Alcoholism	311	0.61	1,179	0.58	0.398	
≧3 visits	CKD	2,579	5.09	9,998	4.93	0.146	
≧3 visits	Migraine	788	1.56	1,573	0.78	< 0.001	
≧3 visits	Osteoporosis	597	1.18	2,341	1.15	0.659	
≧3 visits	Hyperlipidemia	2,839	5.60	11,146	5.50	0.361	
	Autoimmune disease	241	0.48	858	0.42	0.112	

P: Chi-square / Fisher exact test on category variables

Supplementary Table S7. Differences in incidence of injuries in patients with ≥ 1, ≥ 2, and ≥ 3 times of BPPV diagnosis

**BPPV**	**With BPPV**				**Without BPPV** (4-fold matched)				**With *vs.* Without** *(Reference)*			
**BPPV Diagnosis by otolaryngologist or neurologist**	**Populations**	**Events**	**PYs**	**Rate (per 10^5 PYs)**	**Populations**	**Events**	**PYs**	**Rate (per 10^5 PYs)**	**Adjusted HR**	**95% CI**	**95% CI**	***P***
BPPV coding visits ≧ 3	50,675	13,215	709,956.75	1,861.38	202,700	34,421	3,014,434.81	1,141.87	2.63	2.49	2.88	< 0.001
BPPV coding visits ≧ 2	70,613	17,965	989,287.24	1,815.95	282,452	48,024	4,215,860.73	1,139.13	2.47	2.33	2.71	< 0.001
BPPV coding visits ≧ 1	81,310	18,272	1,039,875.63	1,757.13	325,240	53,121	4,753,982.50	1,117.40	2.04	1.90	2.28	< 0.001

BPPV: benign paroxysmal positional vertigo; PYs = Person-years; Adjusted HR = Adjusted Hazard ratio: Adjusted for the variables listed in Table 3.; CI = confidence interval

Supplementary Table S8. Differences in subtypes of injuries between patients with and without BPPV.

BPPV	Total		With BPPV		Without BPPV		*p-value**
**Variables**	**n**	**%**	**n**	**%**	**n**	**%**	
**Total**	253,375		50,675	20.00	202,700	80.00	
**Injury**							< 0.001
Without	205,739	81.20	37,460	73.92	168,279	83.02	< 0.001
With	47,636	18.80	13,215	26.08	34,421	16.98	< 0.001
**Subgroup of injury**							< 0.001
**Unintentional injury**	*31,758*	*66.67*	*9,346*	*70.72*	*22,412*	*65.11*	< 0.001
Traffic injuries	7,320	15.37	2,308	17.47	5,012	14.56	0.001
Poisoning	905	1.90	201	1.52	704	2.05	0.630
Falls	12,667	26.59	4,235	32.05	8,432	24.50	< 0.001
Burns and fires	83	0.17	18	0.14	65	0.19	0.965
Drowning	14	0.03	2	0.02	12	0.03	0.994
Suffocation	182	0.38	40	0.30	142	0.41	0.921
Crushing / cutting / piercing	148	0.31	33	0.25	115	0.33	0.942
Excessive heat	7	0.01	1	0.01	6	0.02	0.998
Injury caused by animal	97	0.20	18	0.14	79	0.23	0.941
Electric current injury	0	0.00	0	0.00	0	0.00	-
Other unintentional injuries	10,335	21.70	2,490	18.84	7,845	22.79	< 0.001
**Intentional injury**	*209*	*0.44*	*29*	*0.22*	*180*	*0.52*	0.828
Suicide	118	0.25	17	0.13	101	0.29	0.906
Homicide / abuse	91	0.19	12	0.09	79	0.23	0.922
**Intention unknown**	22	0.05	4	0.03	18	0.05	0.987
**Without E-Code**	15,647	32.85	3,836	29.03	11,811	34.31	< 0.001

Supplementary Table S8. Continued.

BPPV	Total		With BPPV		Without BPPV		*p-value**
**Variables**	**n**	**%**	**n**	**%**	**n**	**%**	
**Injury Severity Score**							< 0.001
ISS**<16	44,671	93.78	12,500	94.59	32,171	93.46	< 0.001
ISS ≥16 (Major trauma)	2,965	6.22	715	5.41	2,250	6.54	0.278

*****_P_: Chi-square/Fisher's exact test on categorical variables; proportional test on percentage

**ISS: Injury Severity Score
